# Exploring patients’ treatment journeys following randomisation in mental health trials to improve future trial conduct: a synthesis of multiple qualitative data sets

**DOI:** 10.1186/s13063-017-2030-4

**Published:** 2017-06-15

**Authors:** Katrina M. Turner, John Percival, David Kessler, Jenny Donovan

**Affiliations:** 1 School of Social and Community Medicine, University of Bristol, Bristol, UK; 2 The National Institute for Health Research Collaboration for Leadership in Applied Health Research and Care West (NIHR CLAHRC West) at University Hospitals Bristol NHS Foundation Trust, Bristol, UK

**Keywords:** Qualitative research, Clinical trials, Depression, Secondary analysis, Data synthesis

## Abstract

**Background:**

The way in which pragmatic trials are designed suggests that there are differences between the experiences of participants randomised to usual care and intervention arms. These potential differences relate not only to which treatment participants receive but also how they access and engage with their allocated treatment. Such differences could affect trial results. The aim of this study was to assess whether such differences exist and, if they do, to consider their implications for the design of future trials.

**Methods:**

Interview transcripts were sampled from data sets gathered during three qualitative studies, all of which had been nested within large, primary care depression trials. Each study had explored trial participants’ views and experiences of treatments received following randomisation. Transcripts from 37 participants were purposefully sampled, 20 of which were from interviews held with individuals allocated to receive usual GP care. Data were analysed thematically.

**Results:**

There was evidence of differences between trial arms across all three data sets. Intervention participants were willing and able to engage with the treatment to which they had been allocated. Randomisation had led to them embarking upon a clear treatment pathway and receiving care in a context where they felt comfortable discussing their mental health and had sufficient time to do so. Intervention participants also had continuity with and confidence in the practitioners they saw. A few usual-care participants talked about having continuity with and confidence in their GPs. However, most of the usual-care participants reported a reluctance to consult GPs about mental health, difficulties in securing treatment appointments, and little or no changes in care following randomisation. Additionally, most reported a lack of continuity of care and a lack confidence in the treatment available to them.

**Conclusions:**

There are important differences between usual-care and intervention arms that go beyond treatment received, and they relate to how participants experience accessing and engaging with their allocated care. As these differences could affect trial results, researchers may want to measure or reduce them in order to fully appreciate or control for the range of factors that might affect treatment outcomes.

## Background

Randomised controlled trials are viewed as the most appropriate research design for evaluating the effectiveness of health-care interventions [1]. Clinical trials are usually pragmatic in nature, i.e. they measure the benefit treatments produce in routine clinical practice [2], and often aim to assess the effectiveness of a new or modified treatment against ‘treatment as usual’.

Pragmatic trials often evaluate complex interventions (i.e., interventions that include several interacting components) [3]. Guidance on the development and evaluation of complex interventions [4] emphasises various phases which should be undertaken prior to full evaluation within a trial. These phases include reviewing existing evidence, developing a theoretical understanding and conducting feasibility and pilot studies. Following such guidance should result in the intervention being clearly defined prior to the trial starting.

There is less guidance on how to establish the usual-care arm of a trial, and this arm is often poorly defined [5, 6]. Furthermore, whilst usual care is often unrestricted ‘treatment as usual’, researchers may choose to prescribe exactly which treatment(s) individuals in the usual-care arm will receive [7]. Dawson et al. [8] argued that how researchers define usual care should be informed by factors such as the research question, current evidence and what variability exists within current practice.

When interpreting trial results, researchers tend to focus primarily on what treatment participants in different trial arms received, in terms of type and amount (see, e.g., [9–12]). This focus is very narrow. Treatment is a process, and patients’ experiences of accessing and receiving care could also influence their treatment outcomes and thus the trial’s results. This is because a patient’s treatment experiences can influence the extent to which the patient engages with treatment [13]. It is important that trialists acknowledge this because the way in which trials are designed suggests there are variations between the experiences of patients randomised to intervention and usual-care arms regarding factors such as support to access treatment. If this is the case, to appropriately evaluate an intervention and to consider the range of factors that might explain outcome differences between trial arms, researchers need to appreciate how patients set about accessing and engaging with the care to which they have been allocated.

Research funders continue to view mental health research as a priority area [14]. It is predicted that depression and anxiety will be the leading causes of disability in high-income countries by 2030 [15]. In the United Kingdom, most of this disease burden is managed in primary care. Current interventions include pharmacotherapy and psychological interventions such as cognitive behavioural therapy (CBT). In addition, physical activity may be offered to patients with mild to moderate depression [16], and specific treatments may be available to particular groups of patients; for example, health visitors can provide listening visits to women with post-natal depression. Further research is needed because, despite the availability of different treatments for depression, some patients do not respond to antidepressants [17] or recover despite receiving both pharmacotherapy and psychological intervention [18].

We conducted a secondary analysis and synthesis of data collected during three qualitative studies, all of which were nested within large, multi-centred, pragmatic primary care depression trials. Our aim was to bring together participants’ accounts of their experiences following randomisation in order to assess whether there are differences between the experiences of individuals in different trial arms that researchers may want to consider when designing future trials and evaluating complex interventions.

## Methods

### Data set

The lead author was confident that the three data sets selected for this study would accommodate its aim. KMT had led the original studies and therefore was familiar with each data set. In addition, she was aware that each study had entailed conducting in-depth interviews with trial participants to explore their experiences in the trial and the treatments received.

Data were collected between 2006 and 2011. Participants were interviewed after having completed their primary or final outcome measures within the trial (i.e., between 4 and 12 month post-randomisation). In one of the studies, individuals were interviewed on two occasions (Table 1). The first interview had focused on the participants’ initial trial and treatment experiences. The second interview was held 8 months later and assessed participants’ experiences during the later stages of the trial. In total, the data set consisted of 121 interview transcripts.

**Table 1:** Details of the trials and the nested qualitative studies

Trial	Trial aim	Trial design	Intervention treatment	Patients interviewed^a^
1	Assess the clinical effectiveness and cost-effectiveness of a facilitated physical activity intervention plus usual care, versus usual care alone, for patients with a new episode of depression	Patients randomised to receive a physical activity intervention plus usual care or usual care alone	The physical activity intervention was delivered by physical activity facilitators. It consisted of a maximum of 13 sessions; one 1-h face-to-face session, two 45-minute face-to-face sessions and ten 10- to 20-minute telephone sessions.	Thirty-three trial participants interviewed at 4 months post-randomisation, having completed their primary outcome measures for the trial. Nineteen had been randomised to facilitated activity plus usual care, the rest to usual care only. Twenty-one of the 33 were interviewed again 9 months later.
2	Examine the clinical effectiveness and cost-effectiveness of CBT plus usual care, versus usual care alone, for patients with treatment-resistant depression	Participants randomised to face-to-face CBT plus usual care or usual care only	Face-to-Face CBT was delivered by CBT therapists. Patients were allowed up to eighteen 1-h sessions.	Forty trial participants were interviewed 6 months post-randomisation, having completed their primary outcome measures for the trial. Twenty-six had been randomised to CBT plus usual care and 14 to usual care only.
3	Evaluate the clinical effectiveness of antidepressants compared with general supportive care, and to evaluate the clinical effectiveness and cost-effectiveness of antidepressants compared with listening visits as an initial treatment for post-natal depression	Participants randomised to antidepressants or listening visits but could ‘cross’ treatment arms after 4 weeks post-randomisation	Listening visits were delivered by research health visitors. The visits lasted 1-h each. Women were allowed up to eight visits in total.	Twenty-seven trial participants were interviewed at 44 weeks post-randomisation, having received treatment in the trial. Seventeen had been randomised to initially receive listening visits, and ten had been allocated to initially receive antidepressants.

*CBT* Cognitive behavioural therapy

^a^Trial 1 interviews were held between March and November 2009 and between November 2009 and July 2010. Trial 2 interviews were held between April 2010 and February 2011. Trial 3 interviews were held between November 2006 and June 2007

### Trials

The trials hosting the qualitative studies recruited individuals through general practitioner (GP) surgeries located in the United Kingdom. In each trial randomisation had occurred at the level of the individual, and participants had been allocated to one of two treatment arms. Treatments being evaluated included antidepressants, CBT, facilitated physical activity and listening visits. They were delivered by various individuals—GPs, CBT therapists, physical activity facilitators (PAFs) and research health visitors (RHVs)—and were aimed at different patient groups with depression (i.e., patients with a new episode of depression, patients with treatment-resistant depression and women with post-natal depression).

In two of the trials (trials 1 and 2), individuals were allocated either to an intervention plus usual care or to usual care only. *Usual care* was defined as the care the individual would normally receive from his/her GP. In the third trial, women diagnosed with post-natal depression were randomised to initially receive either antidepressants from their GP or listening visits from an RHV. Women randomised to antidepressants were asked to contact their GP.

### Secondary analysis and synthesis of the data

Data were analysed by two of the authors, one of whom (KMT) was familiar with the material and could provide details about the trials and the context in which data had been collected, and another of whom (JP) was ‘naive’ to the material and able to bring a new perspective to the data sets. Both authors are experienced health service researchers with particular expertise in analysing interview data.

Initially, KMT and JP independently read and re-read a sample of six transcripts to gain an overall sense of the data and to consider how the data should be analysed. These transcripts were purposefully sampled from across the three studies to ensure maximum variation in terms of trial arm and participant gender and age. It was agreed that a thematic approach should be taken because this would enable comparisons to be made across the data sets. Having agreed on this approach, KMT and JP re-read the sampled transcripts and noted possible codes. They then met to discuss their codes and to agree on a coding frame. Once agreement was reached, KMT and JP independently coded the sampled transcripts, then met again to compare their coding and to discuss any discrepancies. This discussion led to new codes being added and existing codes being deleted or defined more clearly. Having finalised the coding frame, they sampled further transcripts using the purposeful approach detailed above. These transcripts, along with the initial six, were imported into NVivo [19] and electronically coded by KMT. This allowed the researchers to then electronically retrieve data pertaining to specific codes and analyse them in detail. To enable comparisons to be made within and across the data sets, using an approach based on Framework analysis [20], KMT summarised the data retrieved in a set of tables. The tables were formatted so that the rows represented each interviewee and columns represented the codes developed. Having summarised the data, KMT and JP scrutinised the content of the table columns to identify central themes and deviant cases. Transcripts were sampled until it was felt that analysing further transcripts would not provide any additional insights; that is, transcripts were sampled until data saturation had been reached.

## Results

Forty-four transcripts were analysed in total. Because 7 of these transcripts were from second interviews with participants whose first interviews had been analysed, we had data from 37 participants (Table 2). Twenty of these participants had been randomised to receive care from their GP, either having been allocated to usual care (trials 1 and 2) or having been randomised to initially receive an antidepressant (trial 3). These participants were viewed as being in the usual-care arm of their trial.

**Table 2:** Participants’ details (*n* = 37)

Trial	
1	15
2	12
3	10
Trial arm	
Intervention	17
Usual care	20
Sex	
Male	12
Female	25
Age, years	
20–29	6
30–39	13
40–49	8
50–59	5
60 and older	5

Analysis of the data indicated differences between trial arms regarding participants’ willingness to engage with their allocated treatment and practitioner, the ease with which they could access care, their treatment experiences and their view of their practitioner. Quotes are provided below to illustrate some of the points made. These quotes are tagged according to the participant’s trial, treatment allocation and assigned identification number. When referring to individuals who delivered care within an intervention arm, the term *therapist* is used.

### Reaction to treatment allocation and willingness to consult

Two ‘intervention participants’ described how they were nervous about starting a new treatment, and some mentioned being concerned that they might not be able to schedule the treatment sessions around their other commitments. Most intervention participants, however, indicated that they were pleased with their allocation and keen to start treatment, describing it as ‘*a really good result*’ (trial 1, intervention, participant 13) and ‘*what my aim was*’ (trial 2, intervention, participant 3). In contrast, many of the usual-care participants said they had been disappointed by their allocation, and they felt that they had missed an opportunity to receive a treatment which would benefit them and would now receive inferior care to individuals in the intervention arm:

*I was gutted [having been allocated to usual care].… I remember leaving and just feeling utter despair. I thought that, genuinely at the time I thought that was my last chance.* (Trial 2, usual care, participant 29)

*It did occur to me that it must be blatantly obvious that the people that are having both [treatments] are going to be better off, because I mean nothing’s changed for me at all, so I mean what’s the point of comparing the two.* (Trial 2, usual care, participant 21)

With the exception of trial 3, where women had been asked to contact their GP to start medication, in the accounts there was very little evidence that individuals randomised to receive usual care had made a GP appointment in response to their allocation. This suggested that in trials 1 and 2, randomisation to usual care had not led to participants seeking additional care or viewing the trial as an opportunity for their current treatment to be reviewed. This may have been because they felt there was no expectation that their behaviour would change, because a few of the usual-care participants talked about being in the control or placebo group. Another reason could be because participants were reluctant to seek help from their GP.

Intervention and usual-care participants in all three trials talked about how they were hesitant to consult their GP about their mental health. Reasons given included previous negative GP consultations, worrying that they would be viewed as ‘*crazy*’ (Trial 2, usual care, participant 29), and being concerned that they would need to initiate a conversation about their mental health. Some patients linked this concern to the fact that they had experienced little continuity in terms of which GP they had seen. This led to patients feeling they would need to explain again how they felt, assuming that if they did consult, there was no guarantee they would see the same GP again, and feeling that they had no relationship with a specific practitioner, which in turn led them to worry that they would waste the practitioner’s time:

*Interviewer: And how did you feel about going to your GP?*

*Participant: I kind of thought I didn’t want to waste their time, because I didn’t have a relationship with my doctor; you’re lucky if you see the same doctor more than twice, so going in and talking to them about it, I felt a bit of a fraud.* (Trial 3, usual care, participant 1)

Within the accounts there was also the suggestion that individuals were hesitant to seek help because there was nothing physically wrong with them:

*It’s not like having a physical illness.… You’re never disbelieved if you’ve got a broken leg; if your brain’s not working, it takes an awful lot of, for somebody like me who doesn’t, I don’t like opening up, or didn’t, didn’t like opening up to people because I thought it would be poo-pooed.* (Trial 2, usual care, participant 24)

Another reason participants gave for not wanting to consult their GP was because they did not want to be prescribed an antidepressant due to concerns about medication dependency, side effects and being stigmatised. Last, it was apparent that some participants were reluctant to consult their GPs because they viewed GPs as generalists who did not know about mental health issues:

*People keep telling me I should actually go to see the doctor again, but I haven’t got a lot of faith in GPs because I know they’re GPs, they’re general practitioners, they don’t know about these things.… [I]t doesn’t do a lot of good seeing your GP really.* (Trial 2, usual care, participant 21)

The difference between participants in different trial arms being willing to engage with their allocated treatment suggested there would be discrepancies between the arms in terms of whether participants accessed care. This situation seemed further exacerbated by the fact that intervention and usual-care participants differed in how easily they had found it to secure an appointment.

### Securing a treatment appointment

Individuals randomised to intervention arms were contacted shortly after randomisation by the therapist who would be responsible for their care, to arrange their initial appointment. When describing their experiences of making further appointments, they described how this had been straightforward; they had been able to contact their therapist directly, either by mobile telephone and/or email address, and in the case of some participants, their therapist had agreed to appointment times that worked around the patient’s other commitments.

Already noted is that some usual-care participants were reluctant to contact their GP. Within the usual-care transcripts, practical barriers were also mentioned. Participants described how their GP practice allowed patients to book only so many days in advance and described how it was often difficult to see a specific GP because that GP only worked part-time or because the GP was popular and therefore fully booked. For individuals who worked, practice opening times and geographical distance were also mentioned:

*I find it very difficult to get to my doctor’s because they don’t open during the times that I can get to them. And because I work 25 miles away, it’s really difficult for me to get in to discuss anything.* (Trial 1, usual care, participant 11)

### Experiences of receiving care

Accounts from most of the intervention participants suggested they had been on a clear care pathway. They talked about booking appointments ahead, regularly seeing their therapist and knowing what care they were able to receive (e.g., number of sessions, when and where). The trials had been designed so that individuals randomised to an intervention arm would have continuity of care in terms of which therapist they saw. As intervention participants talked about receiving care from the same therapist, it was evident that this had occurred. They described this continuity of care as allowing them to build a relationship with their therapists and learn to trust them, revisit ideas and agree on goals to work towards. Continuity of care was also described as creating a sense of partnership and increasing the individual’s commitment to their treatment:

*Interviewer: So it wasn’t a question of just being told, it was something you had to?*

*Participant: It was something I worked through with [PAF’s name]. I didn’t get on with it [exercising] I’d be letting her – letting [PAF’s name] down.* (Trial 1, intervention, participant 4)

Some intervention participants described how, during treatment, they felt their mood improving, that they were gaining a better understanding of their depression and learning new skills to better manage their symptoms. This was evident in all three studies but particularly apparent in the accounts given by individuals in trial 2 who had received CBT. Such developments meant some participants ended treatment feeling positive and more in control:

*I did the course of cognitive behaviour therapy; it altered the way I think about things. I’ve managed to break the cycle of the negative thinking, so I’ve sort of gone from a negative, very depressed person to someone who’s more positive. It’s just learning the techniques and applying them, and it just makes you feel better.… I feel a different person.* (Trial 2, intervention, participant 1)

*If you can get yourself into a situation where you’re exercising regularly, you’ve got the motivation, you’re getting yourself fit, you have a sense of achievement.… [T]his perhaps isn’t the right way to say it, but you cease to be a victim of depression as you are if you’re having active treatment.… It gives you some sort of a sense – a sense of self-reliance and self-confidence and independence.* (Trial 1, intervention, participant 14)

Having a clear treatment end point meant therapists could manage the final session in a way that provided closure and supported the individual in moving on. Intervention participants talked about their therapist reassuring them that they were now better able to manage their symptoms and providing them with suggestions of where they could access further support if necessary (e.g., from their GP, bereavement counselling, mother and baby support centre).

When we focused on the usual-care participants’ accounts, there was very little sense of randomisation having led to them embarking upon a clear care pathway. A few participants did talk about seeing their GP every 4 or 6 weeks, and these individuals also reported seeing the same practitioner and receiving ongoing support, but this arrangement was described as existing prior to trial entry. In addition, the other usual-care participants described rarely seeing their GP and rarely seeing the same GP. It was also apparent that, owing to the availability of repeat prescriptions, this could be the situation even when the individual was on medication and felt he/she was struggling:

*Interviewer: So you tend to just get repeat prescriptions?*

*Participant: Yeah, I don’t even speak to anybody.*

*Interviewer: OK, so when was the last time you consulted your GP face to face?*

*Participant: 4–5 months ago…, and that was a different GP to the one that I saw originally. I haven’t seen the lady that put me on it [medication] originally for a year.*

*Interviewer: OK, and how well would you say you’re coping at the moment?*

*Participant: Not very well.* (Trial 1, usual care, participant 11)

The variation between usual-care participants regarding what continuity of care they had experienced was apparent across the three data sets and between participants involved in the same trial. Thus, it seemed to reflect the nature of service delivery in primary care rather than the nature of usual care within a specific trial, as well as the fact that some participants had formed a relationship with a specific GP and sought only to consult that GP, while others had not.

In terms of treatment provided, most individuals who had consulted their GP had received a 10-minute appointment and had been prescribed an antidepressant or, if they were already on medication, a repeat prescription. The only other treatment mentioned during the accounts which had been offered by a GP was a referral for counselling. Four participants mentioned this treatment, and all four explained that they had needed to wait months before receiving this care.

When describing being prescribed medication, while two individuals mentioned that their GP had explained why medication would be personally beneficial, most usual-care participants remarked that antidepressants had been prescribed because they were a quick and easy way for the GP to deal with their depression:

*I haven’t been given very much in the way of different options by the GP. It was very much being treated by medication, and really counselling has never been mentioned, or any other therapies. I mean, I just feel that when I go into a doctor’s surgery, they have got a limited amount of time to deal with it.… Medication was, I suppose, an easy way of dealing with it.* (Trial 1, usual care, participant 18)

In terms of treatment effect, whilst some participants described medication as stabilising their mood, others stated they were unsure what effect medication had on their mental health. In addition, medication was described being a ‘*crunch*’ (trial 1, usual care, participant 11) and as treating the symptoms of depression rather than the cause. Furthermore, most participants who were on medication at the time of the interview said they had been on the same dose for some time. Such comments gave little sense of individuals feeling that medication had moved them forward in terms of managing their depression.

### View of practitioner

Intervention and usual-care participants who had continuity in terms of the therapist/GP they consulted described them as being good listeners, sympathetic, supportive and empathising with their situation. Therapists and GPs were also described as being knowledgeable about depression, although the former were described as being particularly knowledgeable because of their training and because they specifically worked with patients with depression. Their workload was viewed as providing them with not only knowledge but also understanding, which encouraged participants to talk and placed them in a position of being able to reassure individuals:

*That really did help, to know that she was seeing other ladies like, who were similar to me.… I just genuinely felt I could talk to her because she was somebody that knew where I was coming from.… She said everybody’s different and you mustn’t think that “oh, I’m like this now and that, you know, that I’m never going to get over this because I have seen people get over it”.* (Trial 3, intervention, participant 19)

The context in which care was provided, as well as the perceived role of the therapist, also appeared to encourage intervention participants to discuss their depression. Most intervention participants’ treatment sessions lasted about 1 h, giving participants sufficient time to explain how they were feeling. Intervention participants talked about these sessions as being for them, with their therapist tailoring treatment to their needs and being there to listen and provide advice regarding their depression. This gave a sense of participants viewing these sessions as being about them and their mental health. For example, one individual said ‘*She [the RHV] was really there for me…, specifically for the post-natal depression*’ (Trial 3, intervention, participant 5). Treatment sessions were also described as friendly and informal, and there was the suggestion that individuals felt comfortable confiding in their therapists because they were not medical professionals:

*You know that it was like a therapy session, but it was so informal and friendly.… It makes it easier to discuss things…, whereas if there’s more, you know stricter … more clinical and formal, you feel like you don’t wanna sort of give everything, you want to hold something back.* (Trial 2, intervention, participant 1)

*She [the RHV] was the kind of person that was very friendly, open, she really listened. I felt that like, I felt sort of like a bond, as if not a stranger or she’s like a medical person or anything like that.* (Trial 3, intervention, participant 19)

The idea of intervention participants feeling more able to confide in a non-medical professional was also implied by some participants who commented that they had felt it important that sessions had not been recorded in their medical notes:

*That was another important thing, that things weren’t like shared with my GP … those thoughts and stuff like that, I wouldn’t have like, interviewed so openly.… That was another thing that I felt confident about … was that it’s not going to go out to my health visitor or my GP or the hospital or on my records or something like that.* (Trial 3, intervention, participant 19)

It was evident, though, when we focused on the usual-care participants’ accounts, that some participants viewed their GP as someone they could talk openly to. These participants were those who had continuity in terms of the GP they saw. They talked about their GP being someone who would not judge or view them negatively and as someone who could help them with their depression. Continuity of care appeared to have been important in terms of establishing this situation:

*Interviewer: The next thing is to say, ‘can GPs help with depression?’*

*Participant: I think so, yeah, I think so because I feel like, with my GP, I’ve got a good relationship with him, and I can go to him and I can, I can talk to him because [hesitation] he knows, I don’t have to go into everything that’s happened in my life, and I can go in and just have a bit of rant at him and feel a bit better.* (Trial 2, usual care, participant 10)

In contrast, other usual-care participants who did not have continuity of care detailed how they felt their GP had dismissed their symptoms, had patronised them or had not listened. Participants had found such interactions very upsetting and had responded by registering with a different GP or not seeking help, even though they were aware they needed it:

*It was just something about the doctor, I don’t know. She seemed very patronising.… [S]he made me feel little and stupid and thick, so I thought “oh, I’m not going back to see you again.”… I know I need to see her because I know in myself I’m not feeling right. [This participant had previously attempted suicide.] But then I don’t want to go.… [I]t was just her persona towards me, and I thought, “I can’t, I’m not going to be able to come and talk to you if I’m really bad”, and I knew that straight away.* (Trial 3, usual care, participant 15)

A few usual-care participants also talked about not wanting to tell their GP too much because friends and family used the same practice, which, in their view, raised issues of confidentiality. It was also evident that short treatment appointment times were viewed as curtailing discussion:

*You don’t get much time to do that [talk], it’s not like they’re a person you’re going to have a chat with; you’re in and you’re out, aren’t you?* (Trial 3, usual care, participant 1)

## Discussion

The present study demonstrates that there are important differences between the experiences of participants randomised to different trial arms regarding engagement with and access to their allocated care; the amount of care received; the quality of the practitioner-patient relationship, especially in terms of continuity; and the context in which care is delivered. These differences could affect treatment outcomes between trial arms.

Patients’ treatment expectations can influence the effect of an intervention [21], and patient preferences have been shown to affect treatment outcomes in some trials [22, 23], although authors of a systematic review of trials that incorporated participants’ preferences concluded that intervention preferences have limited impact on their validity [24]. Research has shown that both practitioners and patients may view usual care as substandard to the intervention [25, 26] and that patients have a three-fold preference for psychological over pharmacological interventions [27]. This preference was evident in the present study. In addition, it has been argued that allocation to the ‘wrong’ treatment can lead to ‘resentful demoralisation’ [28], which in turn may result in patients disengaging from their usual treatment or seeking alternative interventions [29]. Whilst there was no evidence of usual-care patients disengaging from treatment or seeking additional support, such resentment may create its own bias by altering the psychological outcomes of participants so that they ‘under-perform’ on certain outcome measures [29]. This may be particularly relevant in depression trials and where self-reported outcome measures are used.

Individuals randomised to usual care needed to initiate contact with a practitioner. Patients are often reluctant to disclose mental health problems to their GP [30, 31], and in the present study such reluctance meant, for some usual-care participants, usual care entailed no change in care or, where treatment had not been initiated pre-trial, no care. In contrast, intervention participants automatically received opportunities for changes and increases in care, and the fact that contact was initiated by the therapist himself/herself probably meant that this care was accessed shortly after randomisation.

In the present study it was evident that intervention participants had experienced continuity of care and viewed this situation as enabling them to build a relationship with their therapist. Such continuity is particularly valued by patients when psychological issues are being discussed [32, 33], and patients with depression who have a constructive relationship with their practitioner are more likely than those who do not to comply with prescribed medication or physical activity programmes, to complete therapy and to experience better treatment outcomes [34–39]. Yet, even if continuity had not been achieved, it was apparent that intervention participants received treatment in a context where there were regular appointments; where participants viewed their therapist as particularly knowledgeable about depression; and where there was time to talk, which in itself can be experienced as therapeutic [13]. It was also a context in which both the practitioner and patient expected mental health to be discussed. This expectation removed the need for patients to initiate a conversation about their mental health, a process which usual-care participants described as discouraging them from consulting a GP. It also removed the possibility that patients felt unsure whether it would be appropriate to disclose depressive symptoms, which can be the case in primary care [30, 40].

There was variation in the extent to which usual-care participants received continuity of care and the extent to which patients were regularly seen and regarding follow-up. In terms of actual treatment provided, though, there was little variation, with usual care being primarily described as consisting of 10-minute appointments and being prescribed an antidepressant. GPs were described as having limited ability and interest in treating depression, and this finding, along with the perception of GPs offering only medication, is in keeping with the results of other studies [30, 41].

The GP consultation was rarely described as a context in which patients felt comfortable discussing their mental health or felt they had time to do so. However, the usual-care participants who had continuity of care felt able to talk to their GP and viewed their GP as someone who could help them. The importance participants placed on how practitioners interacted with them supports evidence that suggests patients with depression value practitioner attributes, such as approachability, more than the time or treatment the practitioner can provide [13]. It probably also relates to the fact that, in the management of depression, the practitioners’ interpersonal skills will form a core part of the treatment provided [41].

Having reviewed and synthesised findings from published qualitative studies describing factors affecting recruitment into depression trials, Hughes-Morley et al. [26] found that the reasons individuals took part in depression trials included wanting to access services that would otherwise not be available to them and believing the trial would be personally beneficial. The differences identified in the present study suggest researchers will struggle to describe or define usual care in a way that will reassure potential trial participants that they will receive a level, quality and consistency of care comparable to that of individuals randomised to the intervention arm. In this situation, it may be that researchers need to accept that clinical equipoise will be difficult to demonstrate and consider trial designs such as waiting-list control trials, in which all trial participants eventually receive the trial intervention, or take into account patient preferences for a particular treatment [42]. It may also be that researchers need to consider whether treatment as usual is an acceptable comparator.

Whilst the usual-care arm of a trial may consist of treatment as usual, as was the case in our study, researchers may choose to define the usual-care arm of their trial as care that adheres to clinical guidelines or is, according to current evidence, the best proven therapy [7, 8]. With research funders requesting patient and public involvement in the design of clinical research, its content may also be influenced by patients’ views on what would be considered acceptable. Researchers may have concerns about characterising and clearly defining usual care because doing so could improve its quality and content, which in turn could reduce the effect size of the intervention [43]. However, defining usual care according to guidelines, evidence and patients’ views would help ensure usual-care participants received a certain standard of care and could improve trial recruitment. It might also guard against researchers who want to demonstrate an intervention effect ignoring, consciously or not, the standards and experiences of care associated with usual care.

Researchers in pragmatic trials aim to evaluate interventions within clinical practice, and therefore the context in which care is provided cannot be separated from the actual intervention itself. However, researchers may want to introduce procedures to reduce some of the differences identified in the present study to ensure they are assessing two treatments (i.e., intervention versus more structured GP care) and to limit the extent to which contextual factors or modes of delivery affect treatment outcomes. Possibilities could include requesting that GP practices contact patients randomised to usual care and offer an initial appointment, and provide continuity of care where possible. Individuals involved in recruiting trial participants, or informing them of their treatment allocation, could present usual care as an opportunity for the individual to have their treatment reviewed. They could also proactively address some of the assumptions and scepticism around care provided in general practice by highlighting that GPs regularly deal with depression and that antidepressants are an effective treatment [44]. They could also mention that, with the establishment of Improving Access to Psychological Therapies (IAPT) programme in 2008, GPs now have greater opportunities to refer patients for psychological interventions for depression. In addition, as suggested above, researchers may want to consider whether to try to standardise usual care. National Institute for Health and Care Excellence [45] guidance on the management of depression in adults could be used to inform this process because it includes clear guidance on which treatment should be offered and when, as well as how regularly patients should be followed. Last, researchers may want to measure variables such as patients’ confidence in their practitioners’ ability to treat depression, as well as the level of continuity of care experienced, to better capture participants’ experiences in different trial arms, and to measure factors that might influence treatment adherence and effectiveness.

### Strengths and limitations

The fact that differences between trial arms were identified across all three data sets increases the confidence with which conclusions can be drawn. However, the data synthesised came from interviews with individuals who were participating in mental health trials that all included non-pharmacological interventions. This could limit the generalisability of the findings because it may have been that these trials attracted patients who held particularly negative views towards medication. In addition, the differences found between the accounts of intervention and usual-care participants were probably heightened by the fact that those in the intervention arms were receiving treatments which actively engaged the participants, required them to work with a therapist and aimed to provide them with new skills and insights, whereas those randomised to usual care received mainly a treatment which entailed little engagement and that patients may have viewed as addressing not the cause of their depression but merely the symptoms [46]. It should also be noted that the interviews conducted required participants to recall their treatment and trial experiences. Thus, their accounts were open to post hoc reconstruction and recall bias and may have been shaped by their overall trial and treatment experiences.

Another limitation is that some of the data were collected 10 years ago. During this period, IAPT services have increased primary care patients’ access to psychological treatments [47], so the provision of non-pharmacological interventions in primary care has changed. However, as indicated in this paper, antidepressants remain the first-line treatment for depression in primary care [48], and prescribing rates for antidepressants continue to rise [49]. Over the last 10 years, there has been increasing interest in trial methodology, as evident in the establishment within this time period of, for example, the Medical Research Council Hubs for Trials Methodology Research and journals such as *Trials*. The design of pragmatic trials therefore may have been refined during this period. Yet, some of the points raised in this paper (e.g., patients viewing usual care as substandard to the intervention) are still being debated by those working in clinical trials and not only in the area of mental health [50]. Additionally, as indicated in the discussion above, some of our findings resonate with those of other studies.

## Conclusions

Differences exist between the experiences of individuals who are recruited to mental health trials and allocated to different treatment arms, in terms accessing treatment, the level of attention and consistency they receive, and what treatment and practitioner expectations they have. These differences could affect treatment outcomes of individuals in different trial arms over and above the difference between what is assumed to be standard care and the intervention being tested. Researchers should consider whether they want to introduce processes to reduce some of these differences, whether they want to try to standardise usual care, and/or whether they want to adopt specific trial designs that ensure each trial participant, independent of allocation, feels that he/she benefits from trial participation. Whilst the focus in the present study is on mental health trials, many of the points raised are applicable to trials in other clinical settings.

## Abbreviations

CBT: Cognitive behavioural therapy
GP: General practitioner
IAPT: Improving Access to Psychological Therapies
PAF: Physical activity facilitator
RHV: Research heath visitor

## References

[1] Sibbald B, Roland M (1998). Why are randomised controlled trials important?. BMJ.

[2] Roland M (1998). Understanding controlled trials: what are pragmatic trials?. BMJ.

[3] Ford I, Norrie J (2016). Pragmatic trials. N Engl J Med.

[4] Craig P, Dieppe P, Macintyre S, Michie S, Nazareth I, Petticrew M (2008). Developing and evaluating complex interventions: the new Medical Research Council guidance. BMJ.

[5] Somerville S, Hay E, Lewis M, Barber J, van de Windt D, Hill J (2008). Content and outcome of usual primary care for back pain: a systematic review. Br J Gen Pract.

[6] Erlen JA, Tamres LK, Reynolds N, Golin CE, Rosen MI, Remien RH (2015). Assessing usual care in clinical trials. West J Nurs Res.

[7] Smelt AFH, van der Weele GM, Blom JW, Gussekloo J, Assendelft WJJ (2010). How usual is usual care in pragmatic intervention studies in primary care? An overview of recent trials. Br J Gen Pract.

[8] Dawson L, Zarin DA, Emanuel EJ, Friedman LM, Chaudhari B, Goodman SN (2009). Considering usual medical care in clinical trial design. PLoS Med.

[9] DREAMS Trial Collaborators and West Midlands Research Collaborative (2017). Dexamethasone versus standard treatment for postoperative nausea and vomiting in gastrointestinal surgery: randomised controlled trial (DREAMS trial). BMJ.

[10] Richards DA, Ekers D, McMillan D, Taylor RS, Byford S, Warren FC (2016). Cost and outcome of behavioural activation versus cognitive behavioural therapy for depression (COBRA): a randomised, controlled, non-inferiority trial. Lancet.

[11] Breitenstein C, Grewe T, Floel A, Ziegler W, Springer L, Martus P (2017). Intensive speech and language therapy in patients with chronic aphasia after stroke: a randomised, open-label, blinded-endpoint, controlled trial in a health-care setting. Lancet.

[12] Robling M, Bekkers MJ, Bell K, Bulter CC, Cannings-John R, Channon S (2016). Effectiveness of a nurse-led intervensive home-visitation programme for first-time teenage mothers (building blocks): a pragmatic randomised controlled trial. Lancet.

[13] Percival J, Donovan J, Kessler D, Turner K (2017). *‘She believed in me’.* What patients with depression value in their relationship with practitioners. A secondary analysis of multiple qualitative data sets. Health Expect.

[14] Medical Research Council (MRC). Strategy for lifelong mental health research. London: MRC. 2017. https://www.mrc.ac.uk/documents/pdf/strategy-for-lifelong-mental-health-research/. Accessed 18 May 2017.

[15] Mathers CD, Loncar D (2006). Projections of global mortality and burden of disease from 2002 to 2030. PLoS Med.

[16] National Health Service (NHS). Exercise for depression. 2014. http://www.nhs.uk/Conditions/stress-anxiety-depression/Pages/exercise-for-depression.aspx. Accessed 31 Oct 2016.

[17] Trivedi MH, Rush AJ, Wisniewski SR, Nierenberg AA, Warden D, Ritz L (2006). Evaluation of outcomes with citalopram for depression using measurement-based care in STAR*D: implications for clinical practice. Am J Psychiatry.

[18] Wiles N, Thomas L, Abel A, Ridgway N, Turner N, Campbell J (2013). Cognitive behavioural therapy as an adjunct to pharmacotherapy for primary care based patients with treatment resistant depression: results of the CoBalT randomised controlled trial. Lancet.

[19] QSR International. NVivo Version 10. Doncaster: QSR International.

[20] Ritchie J, Spencer L, Bryman A, Burgess RG (1994). Qualitative data analysis for applied policy research. Analyzing qualitative data.

[21] Bowling A, Ebrahim S (2001). Measuring patients’ preferences for treatment and perceptions of risk. Qual Health Care.

[22] Tilbrook H (2008). Patients’ preferences within randomised trials: systematic review and patient level meta-analysis. BMJ.

[23] Thomas E, Croft PR, Paterson SM, Dziedzic K, Hay EM (2004). What influences participants’ treatment preference and can it influence outcome? Results from a primary care-based randomised trial of shoulder pain. Br J Gen Pract.

[24] King M, Nazareth I, Lampe F, Bower P, Chandler M, Morou M (2005). Impact of participant and physician intervention preferences on randomised trials: a systematic review. JAMA.

[25] Howard L, de Salis I, Tomlin Z, Thornicroft G, Donovan J (2008). Why is recruitment to trials difficult? An investigation into recruitment difficulties in an RCT of supported employment in patients with severe mental illness. Contemp Clin Trials.

[26] Hughes-Morley A, Young B, Waheed W, Small N, Bower P (2015). Factors affecting recruitment into depression trials: systematic review, meta-synthesis and conceptual framework. J Affect Disord.

[27] McHugh RK, Whitton SW, Peckham AD, Welge JA, Otto MW (2013). Patient preference for psychological vs pharmacologic treatment of psychiatric disorders: a meta-analytic review. J Clin Psychiatry.

[28] Cook TD, Campbell DT (1979). Quasi-experimentation: design and analysis issues for field settings.

[29] Torgerson DJ, Torgerson CJ (2008). Designing randomised trials in health, education and the social sciences: an introduction.

[30] Kravitz RL, Paterniti DA, Epstein RM, Rochlen AB, Bell RA, Cipri C (2011). Relational barriers to depression help-seeking in primary care. Patient Educ Couns.

[31] Dew K, Morgan S, Dowell A, McLeod D, Bushnell J, Collings S (2007). ‘It puts things out of your control’: fear of consequences as a barrier to patient disclosure of mental health issues to general practitioners. Sociol Health Illn.

[32] Ridd M, Shaw A, Salisbury C (2006). ‘Two sides of the coin’ — the value of personal continuity to GPs: a qualitative interview study. Fam Pract.

[33] Guthrie B, Wyke S (2006). Personal continuity and access in UK general practice: a qualitative study of general practitioners’ and patients’ perceptions of when and how they matter. BMC Fam Pract.

[34] Weck F, Grikscheit F, Jakob M, Hofling V, Strangier U (2014). Treatment failure in cognitive behavioural therapy: therapeutic alliance as a precondition for an adherent and competent implementation of techniques. Br J Clin Psychol.

[35] Webb CA, DeRubeis RJ, Amsterdam JD, Shelton RC, Hollon SD, Dimidjian S (2011). Two aspects of the therapeutic alliance: differential relations with depressive symptom change. J Consult Clin Psychol.

[36] Searle A, Haase A, Chalder M, Fox KR, Taylor AH, Lewis G (2014). Participants’ experiences of facilitated physical activity for the management of depression in primary care. J Health Psychol.

[37] Gilbert P, Leahy RL (2007). The therapeutic relationship in the cognitive behavioral psychotherapies.

[38] Mellor D, Davison T, McCabe M, George K, Moore K, Chantal S (2006). Satisfaction with general practitioner treatment of depression among residents of aged care facilities. J Aging Health.

[39] Nolan P, Badger F (2005). Aspects of the relationship between doctors and depressed patients that enhance satisfaction with primary care. J Psychiatr Ment Health Nurs.

[40] Prior L, Wood F, Lewis G, Pill R (2003). Stigma revisited, disclosure of emotional problems in primary care consultations in Wales. Soc Sci Med.

[41] Gask L, Rogers A, Oliver D, May C, Roland M (2003). Qualitative study of patients’ perceptions of the quality of care for depression in general practice. Br J Gen Pract.

[42] Brewin C, Bradley C (1989). Patient preferences and randomised clinical trials. BMJ.

[43] de Bruin M, Viechtbauer W, Schaalma HP, Kok G, Abraham C, Hospers HJ (2010). Standard care impact on effects of highly active antiretroviral therapy adherence interventions: a meta-analysis of randomised controlled trials. Arch Intern Med.

[44] Baghai TC, Blier P, Baldwin DS, Bauer M, Goodwin GM, Fountoulakis KN (2011). General and comparative efficacy and effectiveness of antidepressants in the acute treatment of depressive disorders: a report by the WPA section of pharmacopsychiatry. Eur Arch Psychiatry Clin Neurosci.

[45] National Institute for Health and Care Research (NICE). Depression in adults: recognition and management. Clinical guideline [CG90]. London: NICE. October 2009 [last updated April 2016]. https://www.nice.org.uk/guidance/cg90. Accessed 9 June 2017.

[46] Turner KM, Sharp D, Folkes L, Chew-Graham C (2008). Women’s views and experiences of antidepressants as a treatment for postnatal depression: a qualitative study. Fam Pract.

[47] Department of Health. Talking therapies: a 4 year plan of action. London: Department of Health. 2011. https://www.gov.uk/government/publications/talking-therapies-a-4-year-plan-of-action. Accessed 9 June 2017.

[48] Burton C (2012). Antidepressant prescribing – getting it right in primary care. Prescriber.

[49] Prescribing and Medicines Team, Health and Social Care Information Centre. Prescriptions dispensed in the community: England 2004-14. London: NHS Digital. 2015. http://content.digital.nhs.uk/catalogue/PUB17644. Accessed 9 June 2017.

[50] McCambridge J, Sorhaindo A, Quirk A, Nanchahal K (2014). Patient preferences and performance bias in a weight loss trial with a usual care arm. Patient Educ Couns.

